# Matured hiPSC-derived cardiomyocytes possess dematuration plasticity

**DOI:** 10.1016/j.jmccpl.2025.100295

**Published:** 2025-03-28

**Authors:** Fang Meng, Maxwell Kwok, Yen Chin Hui, Ruofan Wei, Alejandro Hidalgo-Gonzalez, Anna Walentinsson, Henrik Andersson, Frederik Adam Bjerre, Qing-Dong Wang, Ditte C. Andersen, Ellen Ngar-Yun Poon, Daniela Später, David C. Zebrowski

**Affiliations:** a Department of Biology, New York University, New York, NY, USA; b Department of Medicine and Therapeutics, Faculty of Medicine, The Chinese University of Hong Kong, Hong Kong SAR, The People's Republic of China; c Hong Kong Hub of Paediatric Excellence (HK HOPE), The Chinese University of Hong Kong, Hong Kong Children's Hospital, Hong Kong SAR, The People's Republic of China; d School of Biomedical Sciences, The Chinese University of Hong Kong, Hong Kong SAR, The People's Republic of China; e Integrated Cardio Metabolic Center (ICMC), Karolinska Institutet, Huddinge, Sweden; f Bioscience Cardiovascular, Research and Early Development, Cardiovascular, Renal and Metabolism (CVRM), BioPharmaceuticals R&D, AstraZeneca, Gothenburg, Sweden; g Murdoch Children's Research Institute (MCRI), Flemington, Melbourne, Australia; h Department of Paediatrics, University of Melbourne, Melbourne, Australia; i Translational Science & Experimental Medicine, Research and Early Development, Cardiovascular, Renal and Metabolism (CVRM), BioPharmaceuticals R&D, AstraZeneca, Gothenburg, Sweden; j Andersen Group, Department of Clinical Biochemistry, Odense University Hospital, Odense, Denmark; k Clinical Institute, University of Southern Denmark, Odense, Denmark; l GenKardia Inc., USA

**Keywords:** Cardiomyocyte, Stem cell, hiPSC, Proliferation, Differentiation, Maturation, Drug discovery, Regeneration, Centrosome

## Abstract

Human induced Pluripotent Stem Cell-derived cardiomyocytes (hiPSC-CMs) are increasingly used to identify potential factors capable of inducing endogenous cardiomyocyte proliferation to regenerate the injured heart. L-type calcium channel blockers have previously been identified as a class of factors capable of inducing matured hiPSC-CMs to proliferate. However, the mechanism by which L-type calcium channel blockers promote hiPSC-CM proliferation remains unclear. Here we provide evidence that matured hiPSC-CMs possess plasticity to undergo dematuration in response to certain pharmacological compounds. Consistent with primary cardiomyocyte maturation during perinatal development, we found that centrosome disassembly occurs in hiPSC-CMs during plate-based, temporal, maturation. A small molecule screen identified nitrendipine, an L-type calcium channel blocker, and 1-NA-PP1, a Src kinase inhibitor, as factors capable of inducing centrosome reassembly in a subpopulation of hiPSC-CMs. Furthermore, centrosome-positive hiPSC-CMs were more likely to exhibit cell cycle activity than centrosome-negative hiPSC-CMs. In contrast, neither nitrendipine or 1-NA-PP1 induced centrosome reassembly, or cell cycle activity, in neonatal rat ventricular myocytes (NRVMs). Differential bulk transcriptome analysis indicated that matured hiPSC-CMs, but not NRVMs, treated with nitrendipine or 1-NA-PP1 undergo dematuration. ScRNA transcriptome analysis supported that matured hiPSC-CMs treated with either nitrendipine or 1-NA-PP1 undergo dematuration. Collectively, our results indicate that matured hiPSC-CMs, but not primary NRVMs, possess plasticity to undergo dematuration in response to certain pharmacological compounds such as L-type calcium channel blockers and Src-kinase inhibitors. This study shows that once mature, hiPSC-CMs may not maintain their maturity under experimental conditions which may have implications for experimental systems where the state of hiPSC-CM maturation is relevant.

## Highlights

• hiPSC-cardiomyocytes are used to identify potential therapeutics for heart failure patients.
• It has been assumed that matured hiPSC-cardiomyocytes maintain their maturity.
• We found that matured hiPSC-derived cardiomyocytes can undergo dematuration.
• In contrast, primary rat cardiomyocytes do not undergo dematuration.
• This can impact the translatability of hiPSC-cardiomyocytes to human disease.

## Graphical abstract

**Figure:**
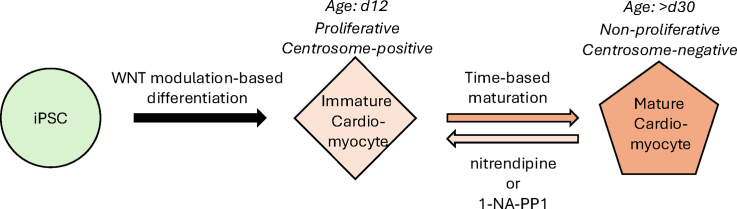

## 1 Introduction

Human induced Pluripotent Stem Cell (hiPSC)-derived cardiomyocytes (hiPSC-CMs) are increasingly used to screen for targets and compounds that have translational therapeutic potential [[1], [2], [3]]. One example for the use of hiPSC-CMs is to identify, or screen for, potential cardiac regenerative factors [4,5]. During perinatal development, cardiomyocytes exhibit a reduction in proliferative activity and ultimately transition to a terminally differentiated state (i.e. an inability to proliferate in response to mitogens) [6]. In comparison to primary postnatal cardiomyocytes, hiPSC-CMs are considered to be ‘fetal-like’ based on their contractile and metabolic behaviors as well as gene expression profiles [7]. However, similar to primary cardiomyocyte maturation during perinatal development, hiPSC-CMs exhibit a reduced ability to proliferate as they mature (e.g. from d12 to d30) [8]. Whether or not hiPSC-CMs truly reach a terminally differentiated state remains unclear, as the process of terminal differentiation in primary mammalian cardiomyocytes has yet to be fully elucidated. Nevertheless, because of age-associated reduction in proliferative potential of primary mammalian cardiomyocytes, matured hiPSC-CMs have been used to identify potential translational targets and factors to induce endogenous human cardiomyocyte proliferation to enable regeneration of the injured human heart [9].

In a previous study, matured hiPSC-CMs (i.e. aged to d36), which exhibit low proliferative activity, were used to identify compounds capable of inducing proliferation [10]. This study identified L-type calcium channel blockers (LTCC) (e.g. nitrendipine and verapamil) as proliferation-inducing, and potentially regenerative, compounds [10]. Why and how LTCCs induce proliferation of matured hiPSC-CMs remains unclear [10]. While LTCCs have not previously been described to regulate native cardiomyocyte proliferation, it has been noted that LTCC blockade suppresses cardiomyocyte differentiation and maturation [[10], [11], [12]]. Furthermore, although a terminally differentiated state has not been explicitly defined, or applied, with regards to hiPSC-CMs, it has been noted that LTCC blockers may enhance hiPSC-CM proliferation, in part, by promoting dedifferentiation (i.e. transitioning from a terminally differentiated-like post-mitotic state to a non-terminally differentiated, proliferation-competent, state) [10].

The terminology of dematuration vs. dedifferentiation is not yet universally agreed upon in the cardiac field (e.g. dedifferentiation could imply the adoption of pre-cardiomyocyte state). During OSKM-overexpression-induced cardiac regeneration, cardiomyocytes have been described as undergoing dematuration largely based upon the restoration of proliferative competency [14,15]. However, cardiomyocyte dematuration without regaining a proliferative-competent phenotype has also been described, as in the case of hypertrophic remodeling in the adult mammalian heart, whereby cardiomyocytes re-express a number of genes predominately expressed within the fetal gene program [16]. Therefore, for purposes of this study, we define cardiomyocyte dematuration as i) a switch from a non-proliferative-competent state (i.e. in which mitogens (e.g. fetal bovine serum) have a limited proliferative effect) to a proliferative-competent state, ii) appearance of a transcriptome signature indicative of a less-mature state, and iii) maintenance of a cardiomyocyte phenotype. Whether or not matured hiPSC-CMs are capable of dematuration, and/or undergo dematuration, remains to be evaluated.

Here, we tested the hypothesis that matured (i.e. >d30) hiPSC-CMs do not maintain their matured state in response to certain pharmacological compounds and can regain the ability to proliferate by undergoing dematuration. In this endeavour, we particularly investigated if the state of centrosome integrity can be utilized as a cellular marker for the state of maturation, or dematuration, in hiPSC-CMs.

## 2 Methods

### 2.1 Antibodies used in this study

Primary antibodies used in this study include: Sarcomeric alpha actinin (1:500 dilution, Abcam, catalogue number: EA-53), Troponin I (1:500 dilution, Abcam, catalogue number: ab56357), Ki67 (1:500 dilution, Abcam, catalogue number: Ab15580), Ki67-488 conjugated (1:500 dilution, BD Pharmingen, catalogue number: 561165), PCM1 (1:500 dilution, Santacruz, catalogue number: sc-H-262), PCM1 (1:500 dilution, Santacruz, catalogue number: sc-398365), CDK5RAP2 (1:500 dilution, Sigma Aldrich, catalogue number: 06-1398), Pericentrin (1:500 dilution, ThermoFisher Scientific, catalogue number: PA5-54109), γ-Tubulin antibody (1:200 dilution, Sigma, catalogue number: T5326). Secondary Antibodies used in this study include: Donkey anti-Mouse IgG (H+L) cross-absorbed secondary antibody, Alexa Flour 488, (1:500 dilution, ThermoFisher Scientific, catalogue number: A-21202), Donkey anti-Rabbit IgG (H+L) cross-absorbed secondary antibody, Alexa Flour 488, (1:500 dilution, ThermoFisher Scientific, catalogue number: A-21206), Donkey anti-Mouse IgG (H+L) cross-absorbed secondary antibody, Alexa Flour 594, (1:500 dilution, ThermoFisher Scientific, catalogue number: A-21203), Donkey anti-Rabbit IgG (H+L) cross-absorbed secondary antibody, Alexa Flour 594, (1:500 dilution, ThermoFisher Scientific, catalogue number: A-21207).

### 2.2 Chemicals used in this study

Cell cycle inhibitors use in this study include Cytosine β-D-arabinofuranoside hydrochloride (araC) (Sigma, catalogue number: C6645) 10 micromolar used in cell culture. Abemaciclib (LY2835219) (Selleckchem, catalogue number: S7158) 1 micromolar used in cell culture.

### 2.3 AstraZeneca compounds used in this study

Compounds used in the small screen include: Repsox: 3.2 micromolar, #6997: 3.2 micromolar, verapamil: 1 micromolar, nitrendipine: 1 micromolar, AZD2691:3.2 micromolar, #4312: 1 micromolar, 1-NA-PP1: 10 micromolar, JTE-907: 3.2 micromolar, #0001: 3.2 micromolar, #7024: 10 micromolar. Compound concentrations were based on those found to optimally achieve hiPSC-CMs proliferation, based upon increases in nuclear count without an increase in multinucleation (personal communication, JJ Saucerman, data not shown). All assays were performed 48 h after treatment of cells with AstraZeneca compounds.

### 2.4 Neonatal rat ventricular cardiomyocyte isolation and culture

Ventricular cardiomyocytes were isolated from 6-day old neonatal Sprague-Dawley rats as described previously (18). Cardiomyocytes were seeded and cultured in DMEM/F-12, Glutamax™-I (ThermoFisher Scientific, catalogue number: 10565018) + Penicillin (100 U/ml)/Streptomycin (100 μg/ml) (Pen/Step) (ThermoFisher Scientific, catalogue number: 15140122) for 2 days prior to experimentation. Cells were plated in 24-well plates on glass coverslips (ThermoScientific. Menzel-Gläser. 13 mm #0, catalogue number: 17274914) coated with Fibronectin for at least 2 h at RT prior to seeding. Treatment of NRVMs with nitrendipine and 1-NA-PP1 was done in the presence of 5%HS and 10%FBS for 2 days prior to fixation or RNA-isolation.

### 2.5 Human iPSC-derived cardiomyocytes and culture

Human induced-PSC-derived cardiomyocytes (hiPSC-CMs) were acquired from Fujifilm Cellular Dynamics International, Inc. (CDI) (iCell Cardiomyocytes, catalogue number: CMC-100-010-001). hiPSC-CMs from CDI were seeded and cultured according to manufacturer's directions (https://cellulardynamics.com/assets/CDI_iCellCardiomyocytes_UG.pdf). CDI iCell Cardiomyocyte lot numbers used in this study: #1833511, #1099441, #1098841. In brief, hiPSC-CMs were thawed and cultured in plating medium (CDI, catalogue number: M1003) for 2 days and then cultured in maintenance medium (CDI, catalogue number: M1001) for 4 days. Afterwards, hiPSC-CMs were treated with compounds for 2 days prior to fixation or RNA isolation. hiPSC-CMs were plated in 24-well plates on glass coverslips (Thermo Fisher Scientific. Menzel-Gläser. 13 mm #0, catalogue number: 17274914) coated with Matrigel (Corning, catalogue number: 356255) for at least 2 h at RT.

### 2.6 AICS-0060-027-derived differentiation and culture

AICS-0060-027-is a human clonal iPS cell line made by the Allen Institute for Cell Science (Cornell Institute). AICS-0060-027 human clonal iPS cell line was differentiated into cardiomyocytes and cultured as described previously, with the exception of being differentiated for 30 days [13,19].

### 2.7 EdU-incorporation assay

EdU-incorporation assay: Six days after seeding hiPSC-CMs, maintenance medium was replaced by fresh maintenance media containing 10 μM 5-ethynyl-2′-deoxyuridine (EdU, ThermoFisher Scientific, catalogue number: C10337) with or without AstraZeneca compounds. Cells were fixed after 2 days and processed for immunofluorescence. Detection of EdU was achieved using Click-iT EdU Alexa Fluor 488 Imaging kit (ThermoFisher Scientific, catalogue number: C10337. (https://tools.thermofisher.com/content/sfs/manuals/mp10338.pdf). Quantification of percentage of centrosome-positive cells that were EdU-positive was achieved by i) first scoring centrosome-positive cells, and then ii) determining how many of these cells are EdU-positive.

### 2.8 Ki67-expression assay

Six days after seeding hiPSC-CMs, maintenance media was replaced by fresh maintenance media containing with or without nitrendipine and 1-NA-PP1. Cells were fixed after 2 days and processed for immunofluorescence analysis. Quantification of percentage of centrosome-positive cells that were Ki67-positive was achieved by i) first scoring centrosome-positive cells, and then ii) determining how many of those cells are Ki67-positive.

### 2.9 Immunofluorescence analysis

Immunohistochemistry was performed as described previously [18]. Images were captured on 1) Zeiss Observer Z1 microscope equipped with Zeiss Axiocam 506 colour and mono cameras, HXP 120 V external light source, Power supply 232, and ZEN 2 (blue edition) imaging software (Zeiss) or 2) Leica DM6B-02 microscope with DFC9000GT camera.

### 2.10 RNA isolation and qRT-PCR

Total RNA was isolated with the TRIzol® Reagent (ThermoFisher Technoloiges) according to the manufacturer's instructions. The quantity and purity of RNA were measured using the Synergy HTX Multi-Mode Reader (BioTek). mRNA was reverse transcribed to cDNA by using Maxima™ H Minus cDNA Synthesis Master Mix Kit, with dsDNase (ThermoFisher Technoloiges) to remove potential DNA contamination. qPCR was performed using SYBR green chemistry, GoTaq® qPCR and RT-qPCR Systems (Promega) and Quant Studio 12K Flex instrument (ThermoFisher Technologies) by following the manufacturer's protocol. Triplicate PCR reactions were performed. Comparative ∆Ct method was used by collecting the mean cycle threshold (Ct) values of triplicate wells for each sample and the expression value was normalized to the endogenous control glyceraldehyde 3-phosphate dehydrogenase (GAPDH). Relative gene expression analysis was determined according to the Thermo Fisher Scientific protocol (https://static.thermoscientific.com/images/D21497∼.pdf). Primers were purchased from ThermoFisher Scientific or Genewiz.

### 2.11 Bulk RNA prep and sequencing

P6-NRVMs and CDI-CMs were treated with nitrendipine or 1-NA-PP1 for 2 days prior to RNA extraction. In addition, both P6-NRVMs and CDI-CMs during treatment with nitrendipine or 1-NA-PP1 were cultured in the presence of araC. AraC was used to reduce overgrowth of contaminating cardiac fibroblasts (which generally amount to ∼3 % of cultured cells in primary cardiomyocyte isolation procedure). RNA was extracted from sub-confluent 10-cm plates using Qiagen RNeasy mini kit. RNA quality (RNA integrity number, RIN) and quantity was measured in a Bioanalyzer 2100 (Agilent) using the Agilent RNA 6000 Nano Kit (part number 5067-1511). The sequencing libraries were prepared using the NEBNext Ultra II Directional RNA Library Prep Kit for Illumina (E7760S, New England Biolabs), starting with 200 ng of total RNA. In brief, mRNA was isolated and fragmented using the NEBNext poly(A) mRNA magnetic isolation module (E7490S, New England Biolabs), with the first and second strands of cDNA synthesized and purified using AmPure XP SPRI beads (A63880, Beckman Coulter). Then adaptor ligation and size selection were performed according to the manufacturer's protocol. Next, the adaptor ligated cDNA was PCR enriched to incorporate an Illumina compatible index sequence (NEBNext Oligos for Illumina, Dual Index Primers Set1 (E7600S, New England Biolabs). All libraries were purified using AmPure XP SPRI beads, and the size distribution of the libraries was analyzed at the Bioanalyzer 2100 using the Agilent High Sensitivity DNA Kit (part number 5067-4626). Subsequently, the quantification of the libraries was performed with the Qubit® 2.0 Fluorometer (ThermoFisher Scientific) and Qubit™ dsDNA HS Assay Kits (Q32851, Invitrogen). Finally, all 19 libraries were pooled and diluted to 3.5 nM for sequencing on one lane of a Hiseq 3000 sequencer (Illumina), using a single read 50 bp and dual indexed sequencing strategy.

### 2.12 Bulk RNA analysis

Downstream functional analysis was performed using Enrichr (https://maayanlab.cloud/Enrichr/) [20] and ShinyGO (http://bioinformatics.sdstate.edu/go/) [21].

### 2.13 scRNA prep and sequencing

D30 AICS-CMs were treated with Condition 1) DMSO (control), Condition 2) nitrendipine, or Condition 3) 1-NA-PP1 for 48 h. This was conducted on two independently differentiated AICS-CMs. AICS-CMs of a respective treated condition were then pooled to give 1 tube of cells, per condition (e.g. cells from both experiments for Condition 1 were pooled), for CDNA prep. Cells were then sent to the CUHK ‘Single Cell Omics Core’ – SCOC – for CDNA prep (https://www2.sbs.cuhk.edu.hk/en-gb/research/core-laboratories/single-cell-omics-core). SCOC performed an initial sample QC check, following by an evaluation of overall quality of sample for fitness to continue the run. Following this, the 10X Genomics protocol was followed. In brief, single cells are then encapsulated in thousands of Gel Bead-in Emulsion (GEM) droplets, where cells are lysed and mRNA molecules reverse transcribed into cDNA. cDNA with specific cell barcodes are then pooled together and subjected to subsequent conventional sequencing library preparation. A final QC is performed on the final sequencing library to ensure sound quality for massively-parallel sequencing. The CDNA was then sent to NovoGene (https://en.novogene.com/services/research-services/pre-made-library-sequencing/) for sequencing. Sequencing Platform & Strategy: NovaSeq PE 150. Data: 10 G of raw data per sample (total of 3 samples). For analysis, NovoGene only conducted data QC.

### 2.14 scRNA analysis

Single-Cell RNA sequencing data analysis was performed as previously described [22]. Briefly, FASTQ files were aligned to the GRCh38 reference genome and counted using the CellRanger platform (version 6.1.1, 10x Genomics). To account for doublets in single-cell isolation, the R-tool DoubletFinder was used, with an assumed doublet formation rate of 7.5 % per sample [23]. Expression matrices were analyzed using standard Seurat V3 pipeline [24]. Low-quality cells were removed from the data by excluding cells that expressed <200 genes and had <25 % reads aligned to mitochondrial genes. For the CM specific analysis, cells that had less than three counts of both TNNT2 and TNNC1 were excluded. Cells were subsequently normalized, scaled and variable features were extracted using the Seurat's built-in command SCTransform. The top 3000 most variable genes were extracted by the Seurat function *FindVariableFeatures* using the variance stabilization transformation selection method and applied for dimensional reduction using principal component analysis (PCA). Subsequently, the top 20 principal components from the PCA analysis were used to compute the Uniform manifold approximation and reduction (UMAP) embedding. Clusters were identified using Louvain clustering at a resolution = 0.1. Comparisons between clusters and treatments, resulting in differentially expressed genes were processed using the Wilcoxon Rank Sum test. GO term analysis were based on the top 100 most differentially expressed genes exhibiting an increased expression for each comparison. GO-enrichment was evaluated using the R-tool ClusterProfiler based on the org.Hs.eg.db database [25]. Cell cycle classification was built on sets of genes made available by Seurat, whereas visualizations were provided using the python package Scanpy and Seurat [26].

### 2.15 Data access

RNA-seq data for scRNA analysis can be accessed at NCBI Gene Expression Omnibus (GEO; https://www.ncbi.nlm.nih.gov/geo/) under accession number PRJNA1049032. RNA-seq data for Bulk analysis can be accessed at NCBI Gene Expression Omnibus (GEO; https://www.ncbi.nlm.nih.gov/geo/) under accession number GSE263350.

### 2.16 Human primers used in this study

**Table 1:** Primer sequences.

Gene	Fwd primer sequence (5'to 3′)	Rev primer sequence (5'to 3′)
*TNNI1*	CAGCTCCACGAGGACTGAAC	CTCTTCAGCAAGAGTTTGCG
*TNNI3*	CCTCAAGCAGGTGAAGAAGG	CAGTAGGCAGGAAGGCTCAG
*MLC2a*	CCGTCTTCCTCACGCTCTT	TGAACTCATCCTTGTTCACCAC
MLC2v	GGAAACCATTCTCAACGCAT	CCTCCTCCTTGGAAAACCTC
*MYH6*	GCTGGCCCTTCAACTACAGA	CTTCTCCACCTTAGCCCTGG
*MYH7*	GAGGACAAGGTCAACACCCT	CGCACCTTCTTCTCTTGCTC
TTN N2B	CCAATGAGTATGGCAGTGTCA	TACGTTCCGGAAGTAATTTGC
TTN N2BA	CAGCAGAACTCAGAATCGA	ATCAAAGGACACTTCACACTC
GAPDH	CGACCACTTTGTCAAGCTCA	GAGGGTCTCTCTCTTCCTCT

### 2.17 Statistical analysis

Data are expressed as the mean ± SEM. Statistical analyses were carried out using a 2-tailed, unpaired Student's *t-*test for 2 groups or 1-way ANOVA followed by Tukey's or Dunnett's test for multiple comparisons. A *P* value of <0.05 was considered statistically significant. All statistical analyses were performed using GraphPad Prism 8 (GraphPad Software).

## 3 Results

### 3.1 The state of centrosome integrity is a cellular marker of hiPSC-CM maturation

**Fig. 1:**
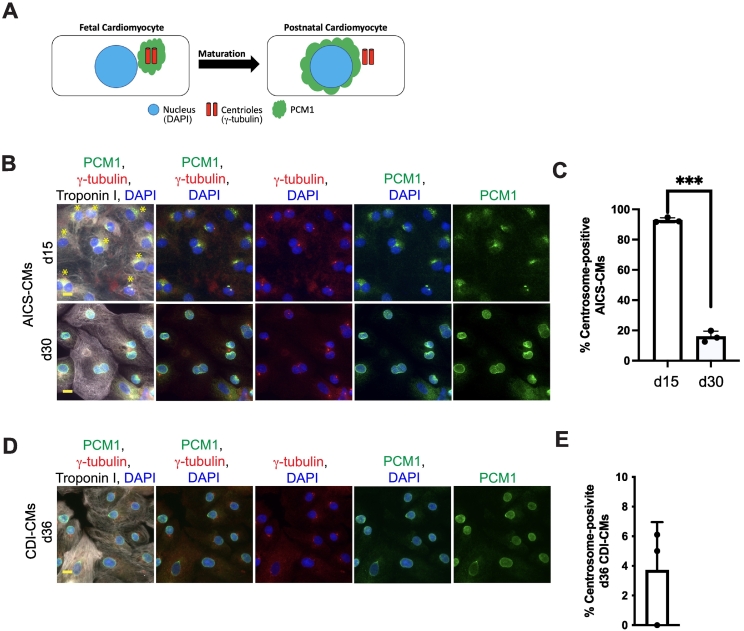

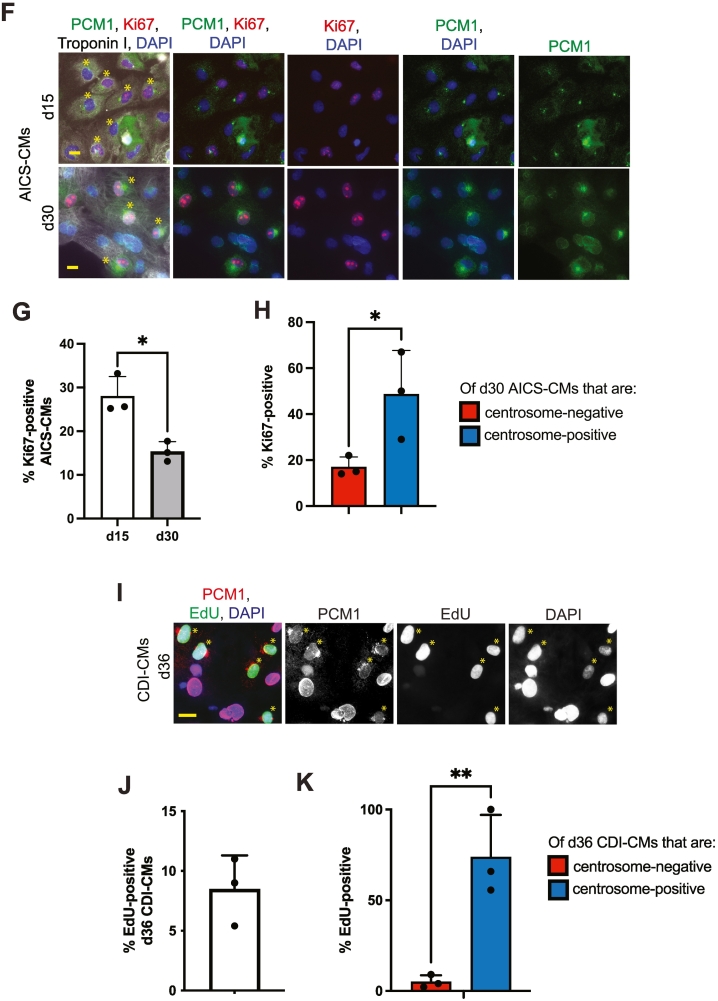
Centrosome integrity reflects the state of maturation in hiPSC-CMs (A) Schematic for centrosome disassembly in cardiomyocytes during perinatal development. (B) Representative images of d15 and d30 AICS-CMs. (C) Quantitation of centrosome-positive AICS-CMs. (D) Images of d36 CDI-CMs. (E) Quantitation of centrosome-positive CDI-CMs. (F) Images of d15 and d30 AICS-CMs. Ki67-positive nuclei indicate AICS-CMs in the cell cycle. Yellow asterisk denotes centrosome-positive hiPSC-CMs. (G) Quantitation of AICS-CMs in the cell cycle. (H) Quantitation of d30 centrosome-positive and centrosome-negative AICS-CMs that are in the cell cycle. (I) Images of d36 CDI-CMs in the cell cycle. Yellow asterisk denotes centrosome-positive hiPSC-CMs. (J) Quantitation of d36 CDI-CMs in the cell cycle. (K) Quantitation of centrosome-positive and centrosome-negative d36 CDI-CMs that are in the cell cycle. Yellow asterisk denotes centrosome-positive hiPSC-CMs. Yellow scale bars = 10 μm. Data are presented as +/− SEM. *P < 0.05, **P < 0.005, ***P < 0.0005, ns = not significant. Statistics were determined using a 2-tailed, unpaired Student's *t-*test. AICS-CM results are from 3 independent experiments from 3 independent differentiations, > 100 cardiomyocytes from 3 different 20× fields were scored per experiment. CDI-CM results are from 3 independent experiments from 3 different lot numbers, > 100 cardiomyocytes from 3 different 20× fields were scored per experiment. (For interpretation of the references to colour in this figure legend, the reader is referred to the web version of this article.)

To measure, and define, the maturation state of a cardiomyocyte, one may include the state of centrosome integrity. The centrosome is a solitary, non-membranous, organelle that is tethered to the nuclear envelope [17]. We have previously reported that the state of centrosome integrity is associated with both maturation and terminal differentiation in primary rat cardiomyocytes. During perinatal development, mammalian cardiomyocytes undergo centrosome disassembly [18]. An early step in centrosome disassembly in mammalian cardiomyocytes during perinatal development is the relocalization of centrosome proteins from the centrioles to the nuclear envelope [18]. For instance, in fetal rat cardiomyocytes, the centriolar satellite protein, Pericentriolar Matrix 1 (PCM1) is associated with the centrioles (Fig. 1A). By birth, however, PCM1 is localized around the nuclear envelope (Fig. 1A) [18]. Thus, by evaluating the localization of PCM1, one may be able to predict the maturation state of cardiomyocytes.

Previously, it has been observed that immature hiPSC-CMs (i.e. ∼d10-d12) have intact centrosomes [27]. However, whether or not centrosome disassembly occurs during maturation in hiPSC-CMs has yet to be evaluated. We differentiated AICS-006-027 hiPSC into cardiomyocytes (AICS-CMs) via the modulation of WNT signalling and assessed the state of their centrosome integrity as they matured. Cardiomyocytes were identified by immunostaining for Troponin I. We identified a cardiomyocyte as being centrosome-positive if PCM1 1) was detected as a punctate signal proximal to the nuclear envelope and 2) colocalized with the centrioles (identified by gamma-tubulin staining). We identified a cardiomyocyte as being centrosome-negative if PCM1 1) was detected as a signal that encircled the nuclear envelope and 2) did not colocalize with the centrioles. We found that the vast majority, ∼90 %, of 15 day (d15) AICS-CMs were centrosome-positive (Fig. 1B and C). In contrast, only ∼18 % of d30 AICS-CMs were centrosome-positive (Fig. 1B and C). We then evaluated the state of centrosome integrity in hiPSC-CMs from FujiFilm Cellular Dynamics International (CDI-CMs). CDI-CMs are cryo-frozen at d30 and then aged another 6 days in culture before analysis. Of d36 CDI-CMs, only ∼4 % were centrosome-positive (Fig. 1D and E). Collectively, these results suggest that, as with primary mammalian cardiomyocytes during perinatal development, centrosome disassembly occurs in hiPSC-CMs during maturation.

In primary rat cardiomyocytes, during centrosome disassembly, PCM1 localization to the nuclear envelope is temporally followed by pericentriolar matrix proteins CDK5RAP2 and Pericentrin (PCNT), in particular the Pericentrin-S isoform. (18). Interestingly, in d36 CDI-CMs, we did not observe CDK5RAP2 or PCNT nuclear envelope localization (S1 Fig. A, B). This suggests that under CDI-CM differentiation conditions (which are proprietary and thus unknown), centrosome disassembly is only partially achieved.

### 3.2 Centrosome-positive hiPSC-CMs preferentially exhibit cell cycle activity

The ability to enter the cell cycle is a feature of immature cardiomyocytes. Given that >d30 hiPSC-CMs showed heterogeneity with regards to centrosome integrity, and that d15 hiPSC-CMs were mostly centrosome-positive, we hypothesized that >d30 centrosome-positive hiPSC-CMs would preferentially exhibit cell cycle activity compared to >d30 centrosome-negative hiPSC-CMs. We found that that cell cycle activity of AICS-CMs was ∼28 % at d15 to ∼17 % at d30, as determined by a Ki67-expression assay (Fig. 1E and G). Furthermore, we observed that cell cycle activity of d30 AICS-CMs preferentially associated with a centrosome-positive (i.e. had a punctate PCM1 signal at the centriole) phenotype, as determined by a Ki67-expression assay (Fig. 1H). Similarly, we observed that cell cycle activity of d36 CDI-CMs preferentially associated with a centrosome-positive phenotype, as determined by a EdU-incorporation assay (Fig. 1J and K). Collectively, these results suggest that 1) > d30 hiPSC-CMs are centrosome-positive are less mature than centrosome-negative hiPSC-CMs and 2) > d30 hiPSC-CMs may exhibit heterogeneity with regards to their maturity.

### 3.3 Identification of compounds that induce centrosome reassembly in hiPSC-CMs

**Fig. 2:**
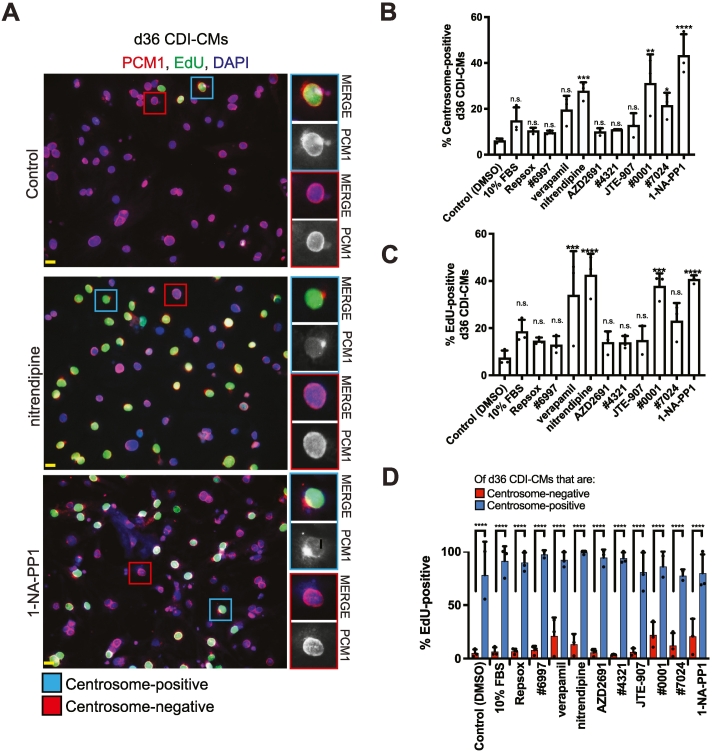

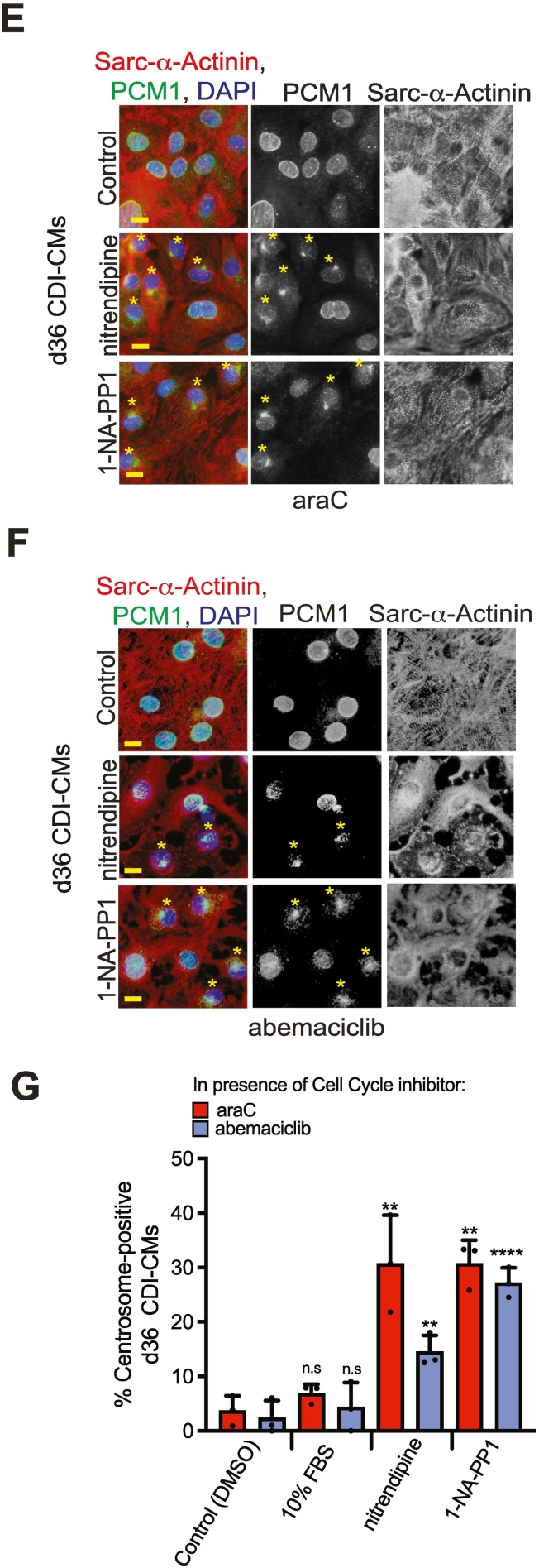
Small molecule screen for hiPSC-CM dematuration factors (A) Representative images of centrosome-positive and centrosome-negative d36 CDI-CMs in EdU-incorporation cell cycle activity assay. Yellow and Red boxes provide examples of centrosome-positive and centrosome-negative CDI-CMs, respectively. (B) Quantitation of centrosome-positive d36 CDI-CMs based on PCM1 location. (C) Quantitation of d36 CDI-CMs in the cell cycle based on EdU incorporation. (D) Quantitation of centrosome-positive and centrosome-negative d36 CDI-CMs that are in the cell cycle. (E) Representative images of centrosome-positive and centrosome-negative d36 CDI-CMs cultures in presence of araC. Yellow asterisks indicate centrosome-positive CDI-CMs. (F) Representative images of centrosome-positive and centrosome-negative d36 CDI-CMs cultures in presence of abemaciclib. Yellow asterisks indicate centrosome-positive CDI-CMs. (G) Quantitation of centrosome-positive d36 CDI-CMs in presence of cell cycle inhibitors compared to Control. Yellow scale bars = 10 μm. Data are presented as +/− SEM. *P < 0.05, **P < 0.005, ***P < 0.0005, ****P < 0.00005, ns = not significant. Statistics were determined using 1-way ANOVA followed by Dunnett's test in (B) and (C) and a 2-tailed, unpaired Student's *t-*test in (D) and (G). CDI-CM results are from 3 independent experiments from 3 different lot numbers, > 100 cardiomyocytes from 3 different 20× fields were scored per experiment. (For interpretation of the references to colour in this figure legend, the reader is referred to the web version of this article.)

Our results indicate that the state of centrosome integrity can be used to evaluate changes in the state of hiPSC-CM maturation. Based on this, we proceeded to use state of centrosome integrity (i.e. relocalization of PCM1 from the centrioles to around the nuclear envelope) and cell cycle activity (i.e. an increase in EDU-incorporation or Ki67-immunostaining) in hiPSC-CMs as markers in a primary screen to identify compounds to determine if any induce hiPSC-CM dematuration. We selected 10 compounds previously identified in a mid-content, 5000 compound, screen based on their ability to increase the number of mononucleated d36 CDI-CMs with a limited increase in the number of binucleated d36 CDI-CMs [10]. Of the 10 compounds tested, 3 compounds significantly increased the percentage of d36 CDI-CMs that were centrosome-positive, with nitrendipine (a dihydropyridine calcium channel blocker [28] and 1-NA-PP1 (a Src kinase inhibitor) [29,30] showing the greatest statistical significance (Fig. 2A, B). Consistent with this result, d36 CDI-CMs treated with nitrendipine or 1-NA-PP1 showed the greatest increase in EdU-positive CDI-CMs (Fig. 2A, C). Furthermore, we found that centrosome-positive d36 CDI-CMs were more likely to be in the cell cycle than centrosome-negative d36 CDI-CMs (Fig. 2A, D). Aging CDI-CMs to d50 did not significantly change the effect of nitrendipine or 1-NA-PP1 on the percentage of centrosome-positive cardiomyocytes (S2 Fig. A, B, and C). Similarly, d50 CDI-CMs treated with nitrendipine or 1-NA-PP1 did not result in a significant change in the percentage centrosome-positive cardiomyocytes in cell cycle (S2 Fig. D, E, F, and G).

Given that an increase in percentage of centrosome-positive hiPSC-CMs occurs within 48 h of nitrendipine or 1-NA-PP1 treatment (see methods), we speculated that the increase in a percentage of centrosome-positive hiPSC-CMs was occurring by way of centrosome reassembly. Nevertheless, although unlikely given the 48 h period of the experiment, the increase in percentage of centrosome-positive hiPSC-CMs could also possibly occur by way of clonal proliferation and expansion of the immature (i.e. centrosome-positive) population. To eliminate the clonal proliferation expansion hypothesis, d36 CDI-CMs were treated with nitrendipine or 1-NA-PP1 and the percent of centrosome-positive cardiomyocytes determined in the presence cell cycle inhibitors. We tested clonal proliferation expansion hypothesis with two different cell cycle inhibitors; abemaciclib (a CDK4/6 inhibitor that suppresses G0/G1 transition) [31] or cytosine arabinoside (araC) (a DNA polymerase inhibitor that suppresses G1/S transition) [32]. To be sure that abemaciclib and araC function as expected, we evaluated their effect on cell cycle progression in d15 AICS-CMs. In the presence of abemaciclib, compared to control conditions (i.e. DMSO), there should be a decrease in the percentage of cardiomyocytes that are Ki67-positive and EDU-positive. In the presence of araC, compared to control (i.e. DMSO), there should be no decrease in the percentage of cardiomyocytes that are Ki67-positive, while there should still be a decrease in the percentage of cardiomyocytes that are EDU-positive (i.e. in presence of araC, entry into the G1 phase of the cell cycle can be attained but not entry into the S phase of the cell cycle). In d15 AICS-CMs, we confirmed that abemaciclib inhibits G0/G1 cell cycle progression and that araC inhibits G1/S cell cycle progression (S3 Fig. A and B). We then tested if centrosome reassembly occurred in d36 CDI-CMs treated with nitrendipine or 1-NA-PP1 in the presence of cell cycle inhibitors for 2 days (i.e. being d38 CDI-CMs at time of analysis). We observed that both nitrendipine or 1-NA-PP1 increased the percentage of centrosome-positive CDI-CMs in the presence of araC (Fig. 2E and G) or abemaciclib (Fig. 2F and G). Furthermore, the increase in percentage of centrosome-positive cardiomyocytes treated with either nitrendipine or 1-NA-PP1 occurred to a similar degree as seen in the absence of araC or abemaciclib (Fig. 1B). Similar to our observations described in Fig. 1, while centrosome-positive hiPSC-CMs were observed under compound treatment, we never observed a centrosome-positive hiPSC-CM that did not have, to some extent, residual PCM1 localization also at the nuclear envelope. This observation may reflect that the process of centrosome reassembly is still progressing in treated hiPSC-CMs or that, multiple, localization-specific, PCM1 isoforms exist (as is the case for Pericentrin) [18]. Nevertheless, collectively, these results indicate that i) the increase in the percentage of centrosome-positive >d30 hiPSC-CMs treated with either nitrendipine or 1-NA-PP1 is due to centrosome reassembly and not clonal proliferation of the centrosome-positive subpopulation and ii), in the case of abemaciclib, centrosome reassembly occurs prior to cell cycle entry.

### 3.4 Primary postnatal cardiomyocytes treated with nitrendipine or 1-NA-PP1 do not exhibit centrosome reassembly or an increase in cell cycle activity

A centrosome-positive phenotype appears to be both a marker of immaturity and proliferative-potential in hiPSC-CMs (shown here), as well as primary mammalian cardiomyocytes [18]. Given that neonatal rat cardiomyocytes exhibit a greater degree of centrosome disassembly than d30+ hiPSC-CMs (i.e. in addition to PCM1 nuclear envelope localization, postnatal cardiomyocytes also exhibit CDK5RAP2 and pericentrin nuclear envelope localization as well as loss of centriole cohesion) we tested if ventricular cardiomyocytes isolated from 6-day (P6) old rat pups (NRVMs) treated with either nitrendipine or 1-NA-PP1 undergo 1) centrosome reassembly or 2) an increase cell cycle activity. The majority of P6 NRVMs are terminally differentiated (i.e. unable to proliferate in response to mitogens), with a minor fraction maintaining the ability to enter a terminal cell cycle [33,34]. Based upon PCM1 localization, neither nitrendipine or 1-NA-PP1 induced centrosome reassembly in P6 NRVMs (S4 Fig. A and B). Furthermore, neither treatment with nitrendipine or 1-NA-PP1 resulted in a significant increase in the percentage of P6 NRVMs in the cell cycle (S4 Fig. C and D). These results indicate that the effects of nitrendipine or 1-NA-PP1 treatment on hiPSC-CMs are not conserved in P6 NRVMs. We speculate that this is because P6 NRVMs are either 1) more mature, and/or 2) have reached a state of centrosome disassembly that is irreversible with nitrendipine or 1-NA-PP1 (and perhaps this is linked to their terminally differentiated state).

### 3.5 Bulk transcriptome analysis indicates that hiPSC-CMs undergo dematuration

Changes in expression of cardiac muscle sarcomere genes is often used to evaluate changes in cardiac maturation [35,36]. We hypothesized that if centrosome-reassembly is a marker of dematuration, then nitrendipine or 1-NA-PP1 should downregulate mature sarcomere gene isoforms. Treatment of d36 CDI-CMs with either nitrendipine or 1-NA-PP1 reduced the expression of sarcomere gene isoforms known to be upregulated during maturation (i.e. MYH7, MLC2v, TNNI3, and TTN-isoform N2B [37]), as determined by qRT-PCR (S5 Fig. A and B). These results indicate that hiPSC-CMs, although already considered immature relative to neonatal rat cardiomyocytes, undergo further dematuration when treated with either nitrendipine or 1-NA-PP1.

**Fig. 3:**
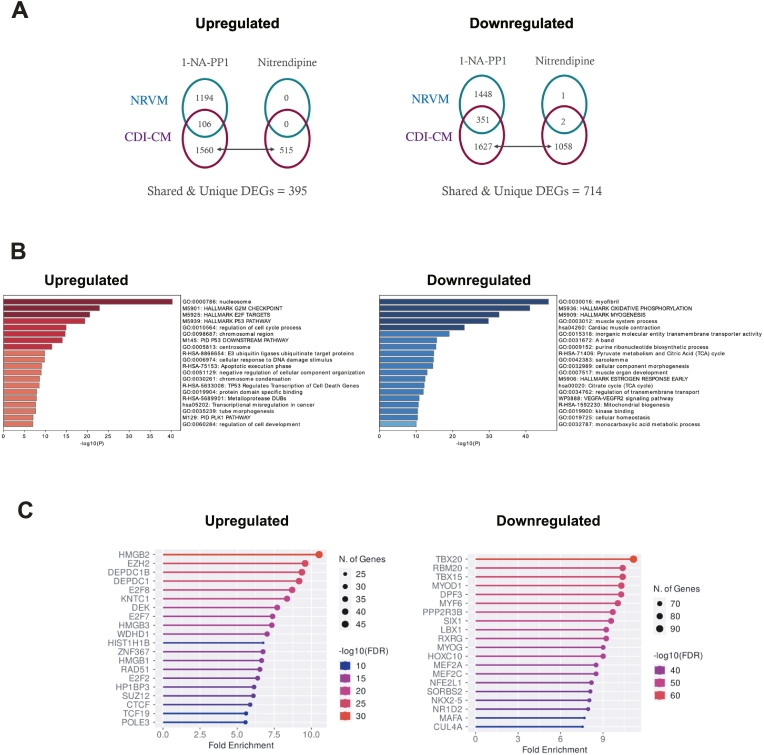
Bulk Transcriptome analysis of hiPSC-CMs and NRVMs (A) Schematic illustrating strategy for differential analysis between hiPSC-CM and P6 NRVM transcriptomes. Shared and Unique refers to genes shared between nitrendipine and 1-NA-PP1 stimulation, and genes unique to hiPSC-CMs (i.e. not expressed in NRVMs). (B) Enriched GO Biological Processes (by Fisher's exact test *P*-value) among common up- and downregulated genes, respectively, specific to hiPSC-CMs treated with either nitrendipine or 1-NA-PP1. (C) Enriched transcription factors (by Fold Enrichment and Enrichment False Discovery Rate) based on co-expression data from the ARCHS4 database (https://maayanlab.cloud/archs4/), among common up- and downregulated genes, respectively, specific to hiPSC-CMs treated with either nitrendipine or 1-NA-PP1.

To further evaluate if dematuration is occurring in hiPSC-CMs, and not occurring in P6 NRVMs, we performed differential bulk transcriptome analysis on both d36 CDI-CMs and P6 NRVMs treated with either nitrendipine or 1-NA-PP1 (Fig. 3A). Unique to CDI-CMs, we found that the top 10 enriched GO Biological Processes among downregulated genes are related to muscle function (including Contraction and Actin-Myosin Filament Sliding) while the top 10 enriched GO Biological Processes among upregulated genes are related to DNA metabolism, Cell Cycle, and Mitosis (Fig. 3B). Furthermore, predictions of upstream transcription factor regulators support that hiPSC-CMs, and not P6 NRVMs, are undergoing dematuration (Fig. 3C). For instance, both HMGB2 and EZH2 were predicted regulators of genes upregulated in hiPSC-CMs. Consistent with this, HMGB2 has previously been shown to be upregulated in OSKM-induced reprogramming of neonatal mouse cardiomyocytes into stem cell-like cardiomyocytes [38] and EZH2 has previously been shown to be down-regulated during embryonic stem cell (ESC)-derived cardiomyocyte maturation [39]. Furthermore, NKX2.5, TBX20, MEF2A and MEF2C were predicted regulators of genes downregulated in hiPSC-CMs. Consistent with this, these factors are progressively upregulated during heart development and/or hiPSC-CM maturation [[40], [41], [42]]. Collectively, bulk transcriptome analysis provides support that hiPSC-CMs, and not P6 NRVMs, undergo dematuration in response to treatment with nitrendipine or 1-NA-PP1.

### 3.6 Single cell transcriptome analysis of hiPSC-CMs treated with nitrendipine or 1-NA-PP1

**Fig. 4:**
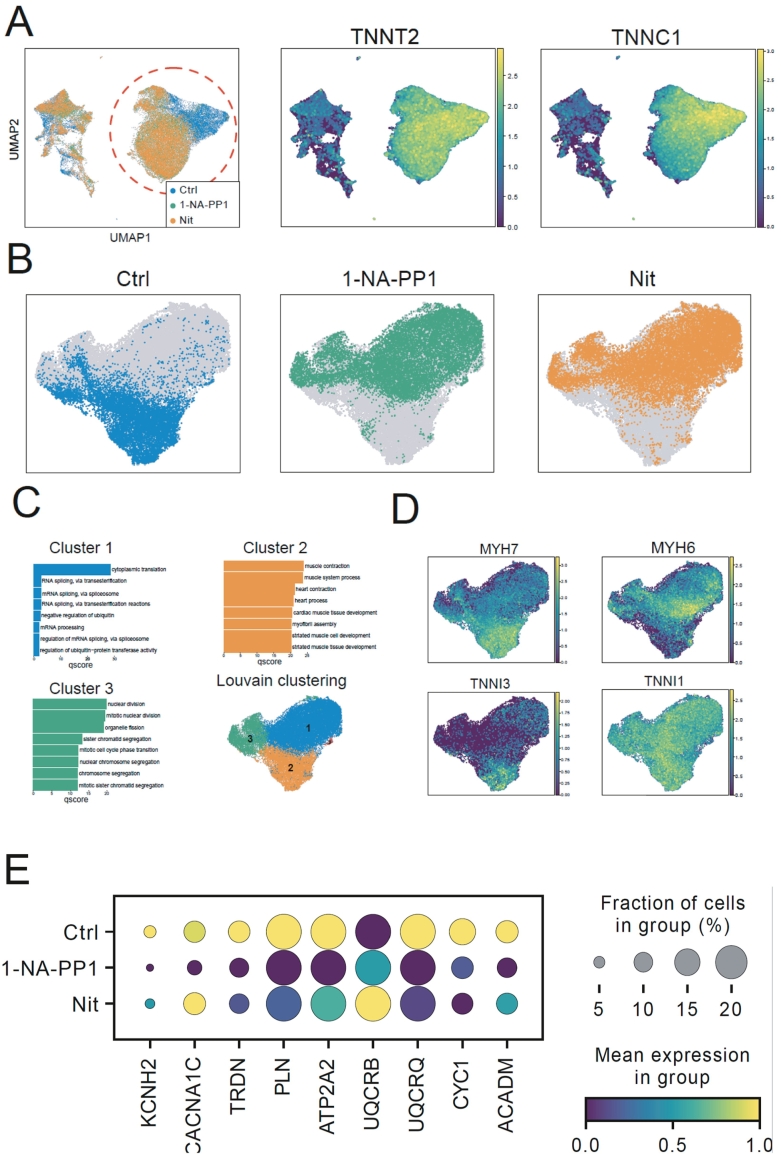
Single Cell RNA sequencing of iPSC-CMs confirms iPSC-CM dematuration. (A) Left: Highly variable gene based low-dimensional UMAP embedding of single cells colored by treatment before filtering. Cells annotated as cardiomyocytes are marked by a dotted circle. Right: scaled log expression of the sarcomere related cardiomyocyte TNNT2 and TNNC1 genes. (B) Highly variable gene based UMAP representation after filtering for hiPSC-CMs based on TNNT2 and TNNC1 expression. (C) Gene Ontology analysis based on top100 differentially induced genes in each cluster for Louvain based clustering of cardiomyocytes. Colors represent different Louvain clusters. qscore represents the -log10 value of adjusted *p*-value for the term. (D) Scaled Log expression of cardiomyocyte maturation associated genes. (E) Dot plots of gene expression for cardiomyocyte maturation markers in control, 1-NA-PP1, and nitrendipine treated hiPSC-CMs. Dot size indicates percentage of cells expression a given gene, and the colour indicates the average log scaled expression level in the cells. Mean expression is visualized using the viridis gradient, transitioning from dark purple indicating low expression towards bright yellow indicating high expression. (For interpretation of the references to colour in this figure legend, the reader is referred to the web version of this article.)

Given that bulk transcriptome analysis indicated that nitrendipine and 1-NA-PP1 induce dematuration in CDI-CMs, we then evaluated if nitrendipine and 1-NA-PP1 induce dematuration in AICS-CMs by scRNA transcriptome analysis. Initially, we filtered the data based on the expression of the cardiomyocyte contractile marker genes TNNT2 and TNNC1 in order to identify the cardiomyocytes in the control, nitrendipine, and 1-NA-PP1 treated cells (Fig. 4A). The filtered data for cardiomyocytes (Fig. 4B-E) were then log-normalized, variable genes identified, and data was scaled prior to clustering and visualization using Uniform Manifold Approximation and Projection (UMAP) plots (Fig. 4B). When merged and integrated as one object, we found that treatment of AICS-CMs treated with 1-NA-PP1 or nitrendipine resulted in a similar shift in clustering when compared to control (i.e. AICS-CMs treated with DMSO) (Fig. 4B). Unsupervised assignment of clusters led to three main cardiomyocyte populations (Cluster 1–3) were identified. Gene Ontology analysis showed that upon treatment with nitrendipine or 1-NA-PP1 hiPSC-CMs adopt an immature (Cluster 1) and proliferative (Cluster 3) gene expression profile (Fig. 4C), whereas control hiPSC-CMs (Cluster 2) mainly exhibited a more mature cardiomyocyte phenotype assigned by GO-terms related to muscle assembly and contraction (Fig. 4C). Thus, the scRNAseq profile of nitrendipine or 1-NA-PP1 treated hiPSC-CMs reflected a state of dematuration, which was supported by the shift of control hiPSC-CMs expressing MYH7 and TNNI1 (i.e. nitrendipine observed in Cluster 2) to nitrendipine or 1-NA-PP1 treated hiPSC-CMs expressing MYH6 and TNNI3 (Fig. 4D). Lastly, we checked differential gene expression for nine cardiomyocyte markers known to be upregulated during maturation [43]. With the exception of UQCRO, all markers had a lower expression in hiPSC-CMs treated with either nitrendipine or 1-NA-PP1 (Fig. 4E). Collectively, these scRNAseq results provide further resolution to the bulk transcriptome data and support that hiPSC-CMs treated with either nitrendipine or 1-NA-PP1 undergo dematuration.

## 4 Discussion

Using the state of centrosome integrity, cell cycle activity, and both bulk and scRNA transcriptome analysis our results suggest that hiPSC-CMs undergo dematuration in response to treatment with nitrendipine or 1-NA-PP1. Given the divergent pharmacological targets of these two compounds, we speculate that hiPSC-CMs can undergo dematuration in response to many other pharmacological compounds. Furthermore, our results, for the first time, suggest that hiPSC-CMs have the plasticity to undergo dematuration.

### 4.1 hiPSC-CM dematuration may explain observations reported in other studies

HiPSC-CMs are increasingly used to determine if therapeutic compounds in preclinical pipeline development are cardiotoxic. Indeed, the American Heart Association has supported the use of hiPSC-CMs for determining cardiotoxicity (e.g. appearance electrophysiological abnormalities, contractile dysfunction, and structural toxicity) for potential cancer therapeutics [44]. Given our observations, it may be of interest to re-evaluate compounds that have previously been identified as being cardiotoxic in hiPSC-CM-based screens. For instance, using hiPSC-CMs, nitrendipine was previously identified as being cardiotoxic in a deep-learning screen aimed at decreasing late-stage drug attrition [45]. Similarly, using hiPSC-CMs, verapamil was previously found to result in reduced gene expression of sarcomere genes (e.g. MYH7 and TNNI3) and cell responses (e.g. reduced calcium kinetics, especially the transient amplitude) [46]. Although we did not investigate verapamil deeply in this study we nevertheless found that verapamil increases the percentage of centrosome-positive d36 CDI-CMs (Fig. 2B). Presuming that the effects of nitrendipine in our study are due to the dematuration plasticity of hiPSC-CMs, we speculate that verapamil's previous effect on reduced expression of sarcomere genes is a result of hiPSC-CM dematuration [46]. Given that neither nitrendipine or 1-NA-PP1 appear to induce dematuration of *primary* cardiomyocytes (i.e. NRVMs) suggests the possibility that factors identified as being cardiotoxic at a particular concentration in hiPSC-CMs may not be cardiotoxic in primary human cardiomyocytes. Indeed, one wonders to what extent other compounds have been deemed to be cardiotoxic using hiPSC-CM-based toxicity screens. One aspect to consider is that nitrendipine (and verapamil) are L-type Ca2+ channel blockers lead to a concentration-dependent increase of beat frequency, accompanied by a reduced contraction force and an eventual stop of contractility at high concentrations (10 μM) (data not shown). This poses the question whether this functional effect/reduction in Ca2+ flux may drive dematuration of iPSC-CMs. 1-NA-PP1 treatment of hiPSC-CMs reduced the beat frequency at higher concentrations (3-10 μM); however, the contraction force remains unaffected based on a surrogate readout (amplitude peak) (data not shown). 1-NA-PP1 does not primarily target calcium handling in cardiomyocytes, but it cannot be excluded that there is an indirect effect. Nevertheless, as nitrendipine and 1-NA-PP1 have rather opposite effects on contractility and a different mode of action, we assume that effects on calcium handling are not the main driver of the observed dematuration, but additional studies would be required for a final clarification.

### 4.2 Centrosome integrity as a marker for better maturation protocols

Native (i.e. primary) postnatal cardiomyocytes are generally described as being ‘more mature’ than native fetal cardiomyocytes (i.e. they exhibit altered contractile properties, metabolic profiles, etc.). Furthermore, while fetal cardiomyocytes are highly proliferative, postnatal cardiomyocytes are terminally differentiated (i.e. unable to proliferate in response mitogenic stimuli). While a number of cardiac pathological conditions (i.e. aortic stenosis, myocardial infarct, etc.) can induce postnatal cardiomyocytes to exhibit features characteristic of de-maturation (e.g. the re-expression of a ‘fetal gene program’ and shifting to a fetal-like metabolic profile) they do not induce a reversal of their terminally differentiated state (i.e. cardiomyocytes to not regain the ability to proliferate). This observation suggests while the processes of cardiomyocyte maturation and terminal differentiation are developmentally correlated across a number of mammalian species that they may be mechanistically independent. Whether or not matured hiPSC-CMs under maturation protocols used here enter a terminally differentiated state remains unclear. However, given that centrosome disassembly was not complete in our matured hiPSC-CMs (i.e. 1) pericentrin does not appear to localize to the nuclear envelope in d30 AICS-CMs or d36 CDI-CMs and 2) loss of centriole cohesion was not observed by qualitative analysis), we speculate that while hiPSC-CMs exhibit reduced proliferative capacity as they mature, that they do not achieve terminal differentiation. It would be interesting to identify maturation protocols that achieve full centrosome disassembly and determine if hiPSC-CMs achieve a terminally differentiated state (i.e. lose plasticity to undergo dematuration and/or regain proliferative ability). For instance, activating the Hippo pathway with compound 19 (C19) has been shown to promote pericentrin localization at the nuclear envelope and loss of centriole cohesion in immature (i.e. ∼ d12) hiPSC-CMs [27]. Similarly, C19 reduces the proliferative capacity of immature hiPSC-CMs [27]. It would interesting to evaluate if hiPSC-CMs treated with C19 lose plasticity to undergo dematuration (and centrosome reassembly) in response to nitrendipine or 1-NA-PP1. If C19-treated hiPSC-CMs do lose the ability to undergo nitrendipine- or 1-NA-PPI-mediated dematuration, then this may be one means to generate, and define, terminal differentiation in hiPSC-CMs. Furthermore, if protocols are developed to induce terminal differentiation in hiPSC-CMs, then they may be a preferred means to generate not only mature hiPSC-CMs, but hiPSC-CMs that are unable to undergo dematuration, particularly with regards to their use in screens for therapeutic or cardiotoxic factors.

## 5 Conclusions

Numerous hiPSC-CM differentiation protocols exist. However, to date and to the best of our knowledge, it has not been evaluated if hiPSC-CMs maintain their maturity under experimental conditions. Here we show that hiPSC-CMs can possess plasticity to undergo dematuration. This has potential consequences with regards to interpreting results in functional, therapeutic, and cardiotoxic experiments and screens. Identifying protocols that make hiPSC-CMs refractory to nitrendipine- or 1-NA-PP1- induced dematuration may prove to be preferrable over existing maturation protocols. Furthermore, our results question to what extent other hiPSC-derived cell-types possess dematuration plasticity, and, if so, how that may affect their application in translational studies.

The following are the supplementary data related to this article.

Fig. S1 Localization of CDK5RAP2 and Pericentrin in hiPSC-CMs. (A) Representative image of CDK5RAP2 localization in d36 CDI-CMs. (B) Representative image of Pericentrin localization in d36 CDI-CMs. Yellow scale bars = 10 μm. Image 1

Fig. S2 Analysis of centrosome integrity and cell cycle activity in d50 hiPSC-CMs. (A) Representative images of centrosome-positive and centrosome-negative d50 CDI-CMs. (B) Quantitation of centrosome-positive d50 CDI-CMs. (C) Quantitative comparison between centrosome-positive d36 CDI-CMs and centrosome-positive d50 CDI-CMs. (D) Representative images of centrosome-positive and centrosome-negative d50 CDI-CMs in Ki67-expression cell cycle activity assay. (E) Quantitation of d50 CDI-CMs in the cell cycle. (F) Quantitative comparison between d36 CDI-CMs in the cell cycle and d50 CDI-CMs in the cell cycle. (G) Quantitation of d50 centrosome-positive CDI-CMs in the cell cycle. Yellow asterisks denote centrosome-positive hiPSC-CMs. Yellow scale bars = 10 μm. Data are presented as +/− SEM. *P < 0.05, **P < 0.005, ****P < 0.00005, ns = not significant. Statistics were determined using a 1-way ANOVA followed by Tukey's test for (B) and (E) and a 2-tailed, unpaired Student's *t* test for (C), (F), and (G). CDI-CM results are from 3 independent experiments from 3 different lot numbers, > 100 cardiomyocytes from 3 different 20× fields were scored per experiment. Fig. S2

Fig. S3 Analysis of cell cycle inhibitors in d15 hiPSC-CMs. (A) Representative images of Ki67-positive and EdU-positive d15 AICS-CMs treated with either araC or abemaciclib. (B) Quantitation of d15 AICS-CM cell cycle activity. Yellow scale bars = 10 μm. Data are presented as +/− SEM. *P < 0.05, **P < 0.005, ns = not significant. n.s. Statistics were determined using a 2-tailed, unpaired Student's *t* test. Results are from 3 independent experiments from 2 independent differentiations. Image 2

Fig. S4 Analysis of centrosome reassembly and cell cycle activity in NRVMs treated with either nitrendipine or 1-NA-PP1. (A) Representative images of cultured cells isolated from P6 rat hearts. NRVMs, which represent >97 % of the culture, are DAPI positive and Sarcomeric alpha actinin positive. Non-cardiomyocytes (e.g. cardiac fibroblasts), which represent <3 % of the culture, are DAPI positive and Sarcomeric alpha-actinin negative. Yellow asterisks indicate centrosome-positive cardiac fibroblasts. (B) Quantitation of centrosome-positive and centrosome-negative NRVMs and non-cardiomyocytes (e.g. cardiac fibroblasts). (C) Representative images of NRVMs in Ki67-expression assay. (D) Quantitation of Ki67 positive NRVMs. n = total cells scored, 2 independent experiments (B) and 3 independent experiments (D). Data are presented as +/− SEM. ns = not significant. Statistics were determined using a 1-way ANOVA followed by Tukey's test. Image 3

Fig. S5 Sarcomere gene expression in hiPSC-CMs treated with nitrendipine or 1-NA-PP1. (A) qRT-PCR analysis of sarcomere gene expression in d36 CDI-CMs treated with nitrendipine. Results from 3 independent experiments. (B) qRT-PCR analysis of sarcomere gene expression in d36 CDI-CMs treated with NA-PP1. Results from 3 independent experiments. Data are presented as +/− SEM. *P < 0.05, **P < 0.005, ***P < 0.0005, ****P < 0.00005, ns = not significant. Statistics were determined using a 2-tailed, unpaired Student's *t* test. Image 4

## CRediT authorship contribution statement

**Fang Meng:** Writing – review & editing, Writing – original draft, Validation, Supervision, Investigation, Formal analysis, Data curation, Conceptualization. **Maxwell Kwok:** Investigation, Data curation. **Yen Chin Hui:** Investigation, Data curation. **Ruofan Wei:** Investigation, Data curation. **Alejandro Hidalgo-Gonzalez:** Investigation, Data curation. **Anna Walentinsson:** Investigation, Formal analysis, Data curation. **Henrik Andersson:** Investigation. **Frederik Adam Bjerre:** Investigation, Formal analysis, Data curation. **Qing-Dong Wang:** Writing – review & editing, Resources, Project administration, Investigation, Conceptualization. **Ditte C. Andersen:** Writing – review & editing, Investigation, Formal analysis, Data curation. **Ellen Ngar-Yun Poon:** Writing – review & editing, Resources, Methodology, Investigation, Formal analysis, Data curation. **Daniela Später:** Writing – review & editing, Writing – original draft, Visualization, Validation, Supervision, Resources, Project administration, Methodology, Investigation, Funding acquisition, Formal analysis, Data curation, Conceptualization. **David C. Zebrowski:** Writing – review & editing, Writing – original draft, Visualization, Validation, Supervision, Resources, Project administration, Methodology, Investigation, Funding acquisition, Formal analysis, Data curation, Conceptualization.

## Institutional review board statement

Extraction of organs and preparation of primary cell cultures were approved by Jordbruks verket, the local Swedish Animal Ethics Committee in accordance to governmental and international guidelines on animal experimentation, permit number S29-15. Ventricular cardiomyocytes were obtained from postnatal day 6 (P6) Sprague–Dawley rats (from Charles River Laboratories, Cologne, Germany or own bred at the (Karolinska Institute, room PKL5).

## Funding

This study was funded by 10.13039/100004325AstraZeneca and Improvement on Competitiveness in Hiring New Faculties Funding Scheme (CUHK) (4930922) to D.C.Z., Hong Kong Research Grants Committee/Early Career Scheme (24118220) to D.C.Z, Hong Kong Research Grants Committee/Early Career Scheme (24100421) to E.P., 10.13039/501100005847Health and Medical Research Fund (HMRF) of Hong Kong (8191456) to D.C.Z and E.P, The 10.13039/501100003554Lundbeck Foundation (#R313-2019-573) to D.C.A., the 10.13039/501100009708Novo Nordisk Foundation (#NNF21OC0071952) to D.C.A, and the Odense University Hospital-Denmark Internationalisation Fund to D.C.A and D.C.Z.

## Declaration of competing interest

D.S., A.W., H. A., and Q.D.W. are employees of AstraZeneca. D.C.Z. was employed by AstraZeneca while conducting a portion of the work for this manuscript. D.C.Z. is no longer affiliated with AstraZeneca. D.C.Z. is an employee of GenKardia. A.H. was employed by AstraZeneca while conducting a portion of the work for this manuscript. A.H. is no longer affiliated with AstraZeneca.

## References

[1] Hnatiuk A.P., Briganti F., Staudt D.W., Mercola M. (2021). Human iPSC modeling of heart disease for drug development. Cell Chem Biol.

[2] Wang P.H., Fang Y.H., Liu Y.W., Yeh M.L. (2022). Merits of hiPSC-derived cardiomyocytes for in vitro research and testing drug toxicity. Biomedicines.

[3] Khan J.M., Lyon A.R., Harding S.E. (2013). The case for induced pluripotent stem cell-derived cardiomyocytes in pharmacological screening. Br J Pharmacol.

[4] Kadota S., Tanaka Y., Shiba Y. (2020). Heart regeneration using pluripotent stem cells. J Cardiol.

[5] Sullivan M.F., Ruemmler P.S., Buschbom R.L. (1986). Influence of iron on plutonium absorption by the adult and neonatal rat. Toxicol Appl Pharmacol.

[6] Zebrowski D.C., Engel F.B. (2013). The cardiomyocyte cell cycle in hypertrophy, tissue homeostasis, and regeneration. Rev Physiol Biochem Pharmacol.

[7] Ottaviani D., Ter Huurne M., Elliott D.A., Bellin M., Mummery C.L. (2023). Maturing differentiated human pluripotent stem cells in vitro: methods and challenges. Development.

[8] Yang H., Yang Y., Kiskin F.N., Shen M., Zhang J.Z. (2023). Recent advances in regulating the proliferation or maturation of human-induced pluripotent stem cell-derived cardiomyocytes. Stem Cell Res Ther.

[9] Fan D., Wu H., Pan K., Peng H., Wu R. (2021). Regenerating damaged myocardium: a review of stem-cell therapies for heart failure. Cells.

[10] Woo L.A., Tkachenko S., Ding M., Plowright A.T., Engkvist O., Andersson H. (2019). High-content phenotypic assay for proliferation of human iPSC-derived cardiomyocytes identifies L-type calcium channels as targets. J Mol Cell Cardiol.

[11] Linask K.L., Linask K.K. (2010). Calcium channel blockade in embryonic cardiac progenitor cells disrupts normal cardiac cell differentiation. Stem Cells Dev.

[12] Puceat M., Jaconi M. (2005). Ca2+ signalling in cardiogenesis. Cell Calcium.

[13] Ahmed R.E., Anzai T., Chanthra N., Uosaki H. (2020). A brief review of current maturation methods for human induced pluripotent stem cells-derived cardiomyocytes. Front Cell Dev Biol.

[14] Beisaw A., Wu C.C. (Jan 2024). Cardiomyocyte maturation and its reversal during cardiac regeneration. Dev Dyn.

[15] Chen Y., Luttmann F.F., Schoger E., Scholer H.R., Zelarayan L.C., Kim K.P. (2021). Reversible reprogramming of cardiomyocytes to a fetal state drives heart regeneration in mice. Science.

[16] van der Pol A., Hoes M.F., de Boer R.A., van der Meer P. (2020). Cardiac foetal reprogramming: a tool to exploit novel treatment targets for the failing heart. J Intern Med.

[17] Lin M., Xie S.S., Chan K.Y. (2022). An updated view on the centrosome as a cell cycle regulator. Cell Div.

[18] Zebrowski D.C., Vergarajauregui S., Wu C.C., Piatkowski T., Becker R., Leone M. (2015). Developmental alterations in centrosome integrity contribute to the post-mitotic state of mammalian cardiomyocytes. Elife.

[19] Kwok M., Lee C., Li H.S., Deng R., Tsoi C., Ding Q. (2022). Remdesivir induces persistent mitochondrial and structural damage in human induced pluripotent stem cell-derived cardiomyocytes. Cardiovasc Res.

[20] Xie Z., Bailey A., Kuleshov M.V., Clarke D.J.B., Evangelista J.E., Jenkins S.L. (2021). Gene set knowledge discovery with Enrichr. Curr Protoc.

[21] Ge S.X., Jung D., Yao R. (2020). ShinyGO: a graphical gene-set enrichment tool for animals and plants. Bioinformatics.

[22] Bak S.T., Harvald E.B., Ellman D.G., Mathiesen S.B., Chen T., Fang S. (2023). Ploidy-stratified single cardiomyocyte transcriptomics map Zinc Finger E-Box Binding Homeobox 1 to underly cardiomyocyte proliferation before birth. Basic Res Cardiol.

[23] McGinnis C.S., Murrow L.M., Gartner Z.J. (2019). DoubletFinder: doublet detection in single-cell RNA sequencing data using artificial nearest neighbors. Cell Syst.

[24] Butler A., Hoffman P., Smibert P., Papalexi E., Satija R. (2018). Integrating single-cell transcriptomic data across different conditions, technologies, and species. Nat Biotechnol.

[25] Wu T., Hu E., Xu S., Chen M., Guo P., Dai Z. (2021). clusterProfiler 4.0: a universal enrichment tool for interpreting omics data. Innovation.

[26] Wolf F.A., Angerer P., Theis F.J. (2018). SCANPY: large-scale single-cell gene expression data analysis. Genome Biol.

[27] Ng D.C.H., Richards D.K., Mills R.J., Ho U.Y., Perks H.L., Tucker C.R. (2020). Centrosome reduction promotes terminal differentiation of human cardiomyocytes. Stem Cell Rep.

[28] Stoepel K., Heise A., Kazda S. (1981). Pharmacological studies of the antihypertensive effect of nitrendipine. Arzneimittelforschung.

[29] Endo S., Satoh Y., Shah K., Takishima K. (2006). A single amino-acid change in ERK1/2 makes the enzyme susceptible to PP1 derivatives. Biochem Biophys Res Commun.

[30] Bishop A.C., Kung C.Y., Shah K., Witucki L., Shokat K.M., Liu Y. (1999). Generation of monospecific nanomolar tyrosine kinase inhibitors via a chemical genetic approach. J Am Chem Soc.

[31] Crozier L., Foy R., Mouery B.L., Whitaker R.H., Corno A., Spanos C. (2022). CDK4/6 inhibitors induce replication stress to cause long-term cell cycle withdrawal. EMBO J.

[32] Kurki P., Vanderlaan M., Dolbeare F., Gray J., Tan E.M. (1986). Expression of proliferating cell nuclear antigen (PCNA)/cyclin during the cell cycle. Exp Cell Res.

[33] Li F., Wang X., Capasso J.M., Gerdes A.M. (1996). Rapid transition of cardiac myocytes from hyperplasia to hypertrophy during postnatal development. J Mol Cell Cardiol.

[34] Zebrowski D.C., Becker R., Engel F.B. (2016). Towards regenerating the mammalian heart: challenges in evaluating experimentally induced adult mammalian cardiomyocyte proliferation. Am J Physiol Heart Circ Physiol.

[35] Bedada F.B., Wheelwright M., Metzger J.M. (2016). Maturation status of sarcomere structure and function in human iPSC-derived cardiac myocytes. Biochim Biophys Acta.

[36] Poon E.N., Luo X.L., Webb S.E., Yan B., Zhao R., Wu S.C.M. (2020). The cell surface marker CD36 selectively identifies matured, mitochondria-rich hPSC-cardiomyocytes. Cell Res.

[37] Guo Y., Pu W.T. (2020). Cardiomyocyte maturation: new phase in development. Circ Res.

[38] Cheng Y.Y., Yan Y.T., Lundy D.J., Lo A.H., Wang Y.P., Ruan S.C. (2017). Reprogramming-derived gene cocktail increases cardiomyocyte proliferation for heart regeneration. EMBO Mol Med.

[39] Kuppusamy K.T., Jones D.C., Sperber H., Madan A., Fischer K.A., Rodriguez M.L. (2015). Let-7 family of microRNA is required for maturation and adult-like metabolism in stem cell-derived cardiomyocytes. Proc Natl Acad Sci USA.

[40] Clowes C., Boylan M.G., Ridge L.A., Barnes E., Wright J.A., Hentges K.E. (2014). The functional diversity of essential genes required for mammalian cardiac development. Genesis.

[41] Tsoi C., Deng R., Kwok M., Yan B., Lee C., Li H.S. (2022). Temporal control of the WNT signaling pathway during cardiac differentiation impacts upon the maturation state of human pluripotent stem cell derived cardiomyocytes. Front Mol Biosci.

[42] Poon E.N., Hao B., Guan D., Jun Li M., Lu J., Yang Y. (2018). Integrated transcriptomic and regulatory network analyses identify microRNA-200c as a novel repressor of human pluripotent stem cell-derived cardiomyocyte differentiation and maturation. Cardiovasc Res.

[43] Ronaldson-Bouchard K., Ma S.P., Yeager K., Chen T., Song L., Sirabella D. (2018). Advanced maturation of human cardiac tissue grown from pluripotent stem cells. Nature.

[44] Gintant G., Burridge P., Gepstein L., Harding S., Herron T., Hong C. (2019). Use of human induced pluripotent stem cell-derived cardiomyocytes in preclinical Cancer drug cardiotoxicity testing: a scientific statement from the American Heart Association. Circ Res.

[45] Grafton F., Ho J., Ranjbarvaziri S., Farshidfar F., Budan A., Steltzer S. (2021). Deep learning detects cardiotoxicity in a high-content screen with induced pluripotent stem cell-derived cardiomyocytes. Elife.

[46] Lam C.K., Tian L., Belbachir N., Wnorowski A., Shrestha R., Ma N. (2019). Identifying the transcriptome signatures of calcium channel blockers in human induced pluripotent stem cell-derived cardiomyocytes. Circ Res.

