# Supplementary figures and images for: Matured hiPSC-derived cardiomyocytes possess dematuration plasticity
Source: J Mol Cell Cardiol Plus. 2025 Mar 28;12:100295. doi: 10.1016/j.jmccpl.2025.100295 (PMC12008595; doi:10.1016/j.jmccpl.2025.100295)

## Supplemental Figure 1

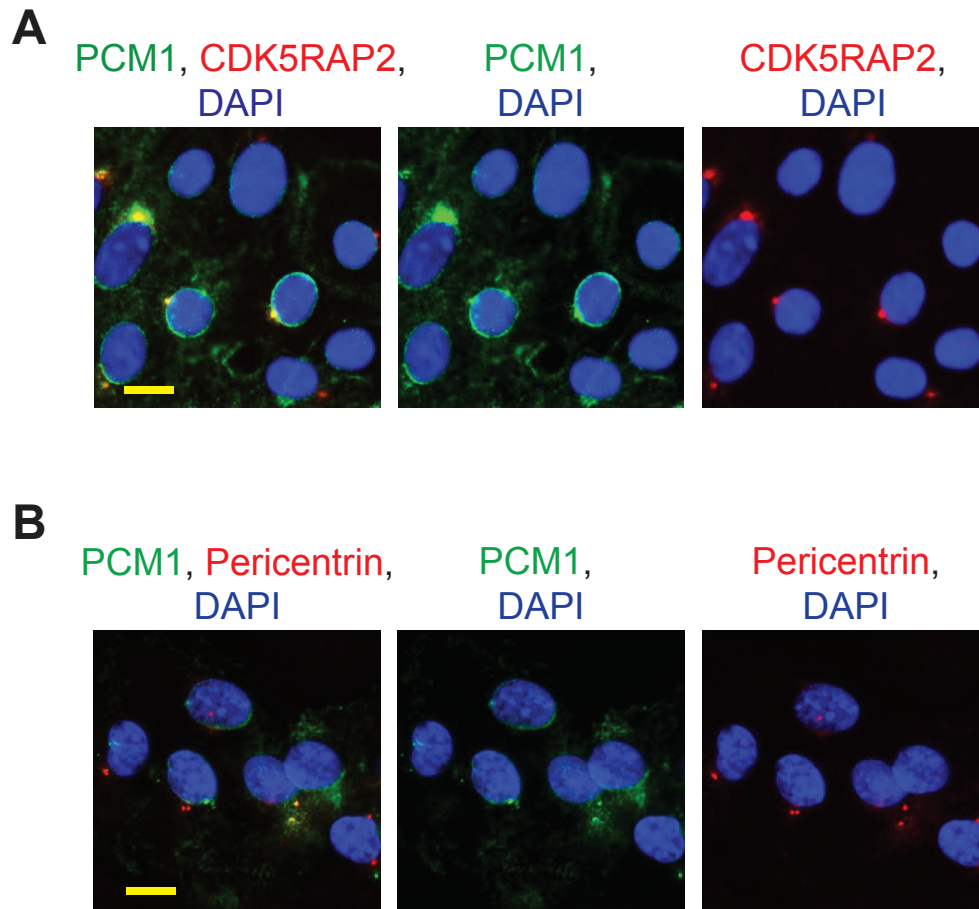

Supplement: Fig. S1 — Localization of CDK5RAP2 and Pericentrin in hiPSC-CMs. (A) Representative image of CDK5RAP2 localization in d36 CDI-CMs. (B) Representative image of Pericentrin localization in d36 CDI-CMs. Yellow scale bars = 10 μm. [file mmc1.pdf]

Supplemental Figure 2

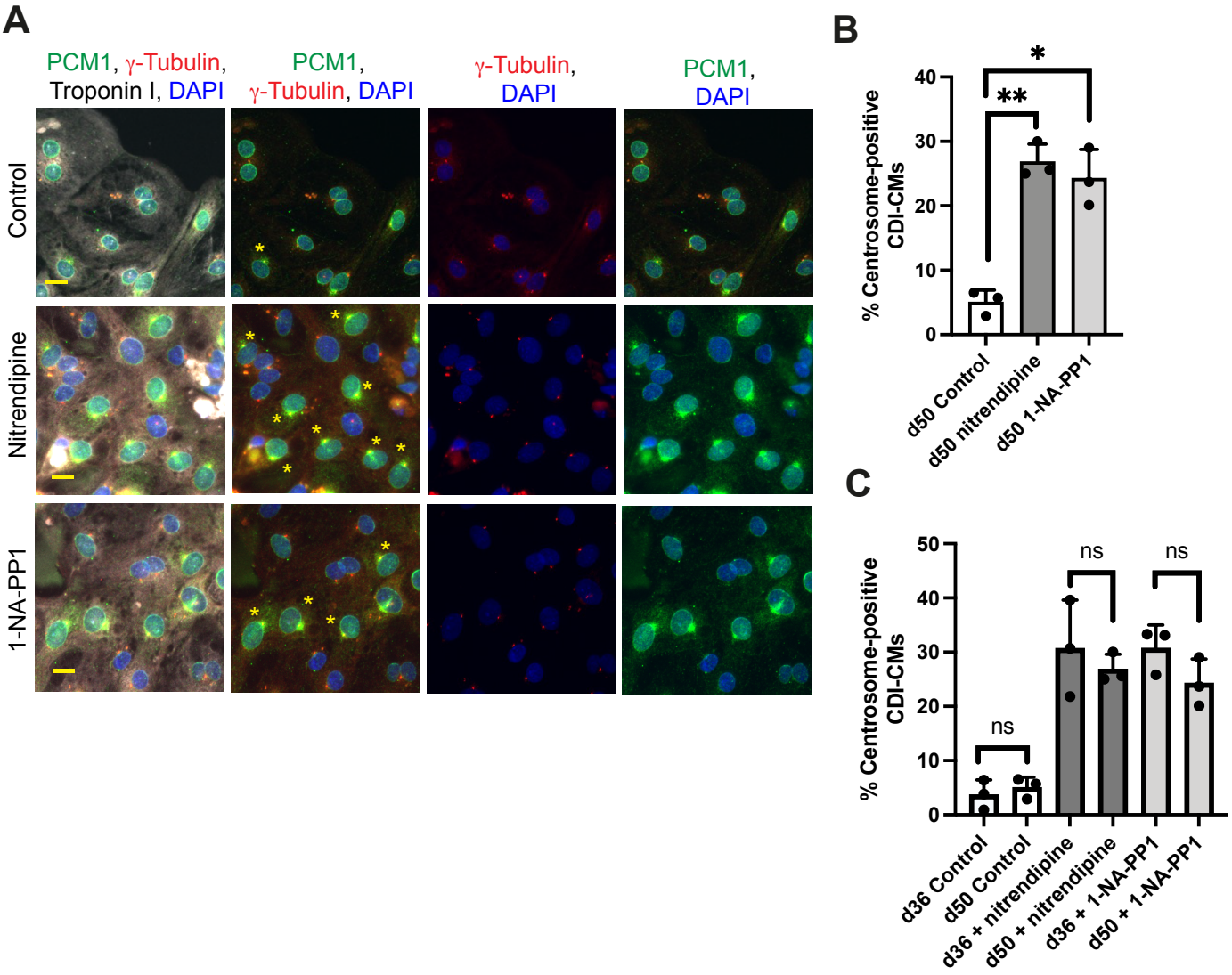

Supplement: Fig. S2 — Analysis of centrosome integrity and cell cycle activity in d50 hiPSC-CMs. (A) Representative images of centrosome-positive and centrosome-negative d50 CDI-CMs. (B) Quantitation of centrosome-positive d50 CDI-CMs. (C) Quantitative comparison between centrosome-positive d36 CDI-CMs and centrosome-positive d50 CDI-CMs. (D) Representative images of centrosome-positive and centrosome-negative d50 CDI-CMs in Ki67-expression cell cycle activity assay. (E) Quantitation of d50 CDI-CMs in the cell cycle. (F) Quantitative comparison between d36 CDI-CMs in the cell cycle and d50 CDI-CMs in the cell cycle. (G) Quantitation of d50 centrosome-positive CDI-CMs in the cell cycle. Yellow asterisks denote centrosome-positive hiPSC-CMs. Yellow scale bars = 10 μm. Data are presented as +/− SEM. *P < 0.05, **P < 0.005, ****P < 0.00005, ns = not significant. Statistics were determined using a 1-way ANOVA followed by Tukey's test for (B) and (E) and a 2-tailed, unpaired Student's t test for (C), (F), and (G). CDI-CM results are from 3 independent experiments from 3 different lot numbers, > 100 cardiomyocytes from 3 different 20× fields were scored per experiment. [file mmc2.pdf]

# Supplemental Figure 2 continued

D

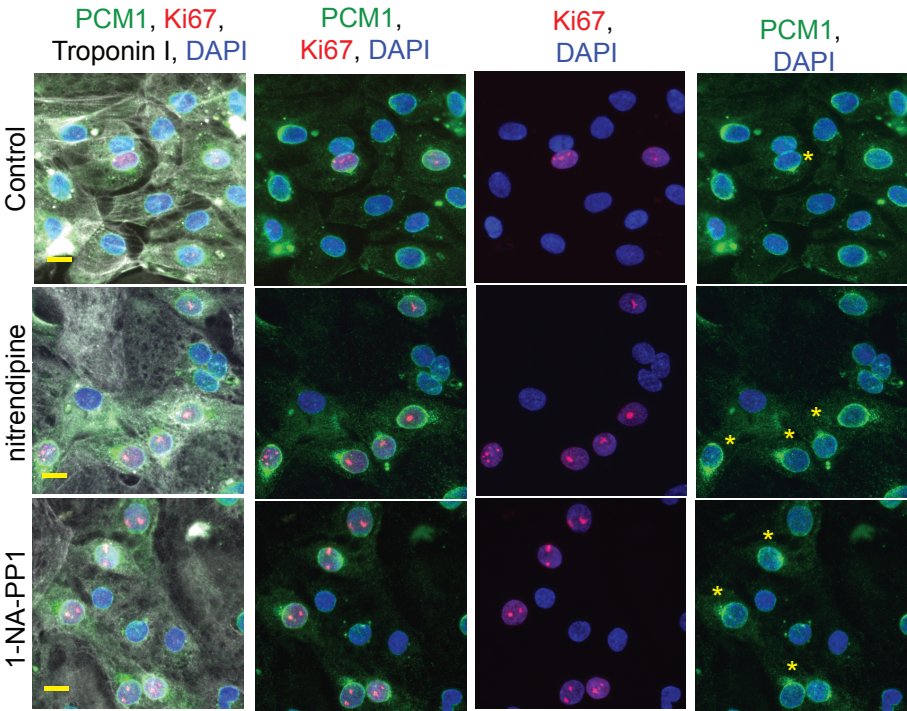

E

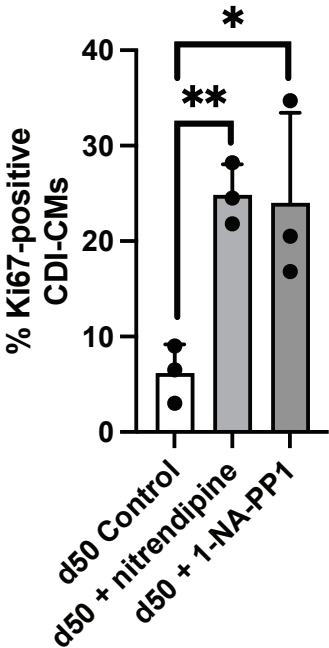

F

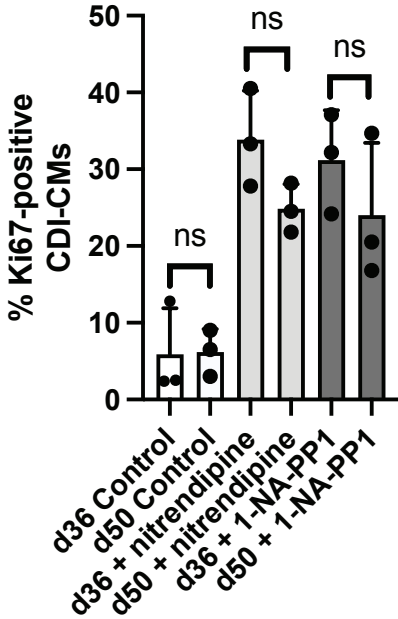

G

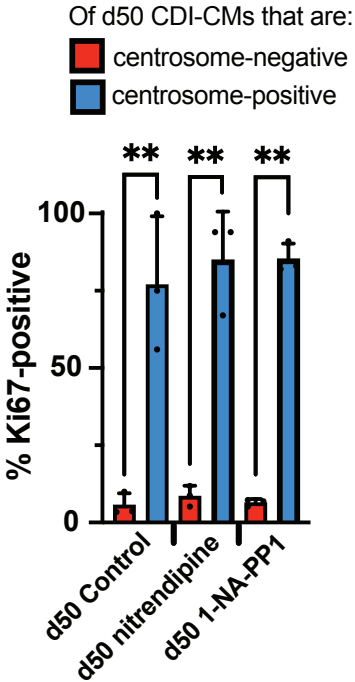

Supplement: Fig. S2 — Analysis of centrosome integrity and cell cycle activity in d50 hiPSC-CMs. (A) Representative images of centrosome-positive and centrosome-negative d50 CDI-CMs. (B) Quantitation of centrosome-positive d50 CDI-CMs. (C) Quantitative comparison between centrosome-positive d36 CDI-CMs and centrosome-positive d50 CDI-CMs. (D) Representative images of centrosome-positive and centrosome-negative d50 CDI-CMs in Ki67-expression cell cycle activity assay. (E) Quantitation of d50 CDI-CMs in the cell cycle. (F) Quantitative comparison between d36 CDI-CMs in the cell cycle and d50 CDI-CMs in the cell cycle. (G) Quantitation of d50 centrosome-positive CDI-CMs in the cell cycle. Yellow asterisks denote centrosome-positive hiPSC-CMs. Yellow scale bars = 10 μm. Data are presented as +/− SEM. *P < 0.05, **P < 0.005, ****P < 0.00005, ns = not significant. Statistics were determined using a 1-way ANOVA followed by Tukey's test for (B) and (E) and a 2-tailed, unpaired Student's t test for (C), (F), and (G). CDI-CM results are from 3 independent experiments from 3 different lot numbers, > 100 cardiomyocytes from 3 different 20× fields were scored per experiment. [file mmc3.pdf]

Supplemental Figure 3

A

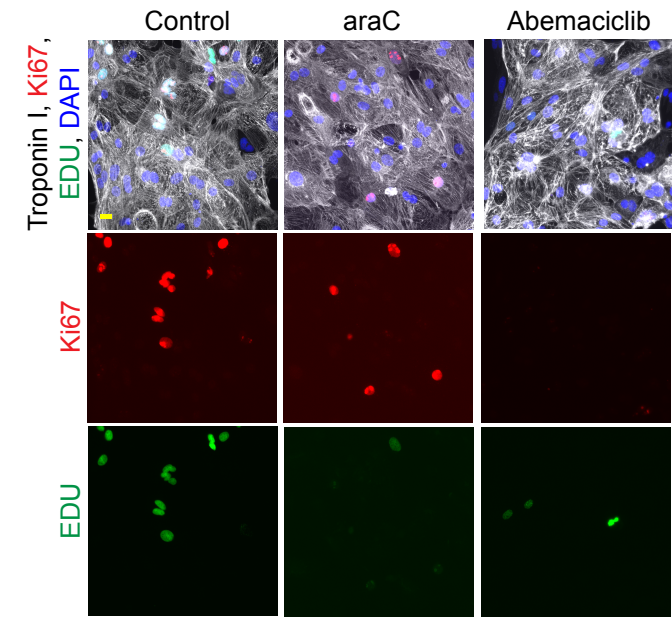

B

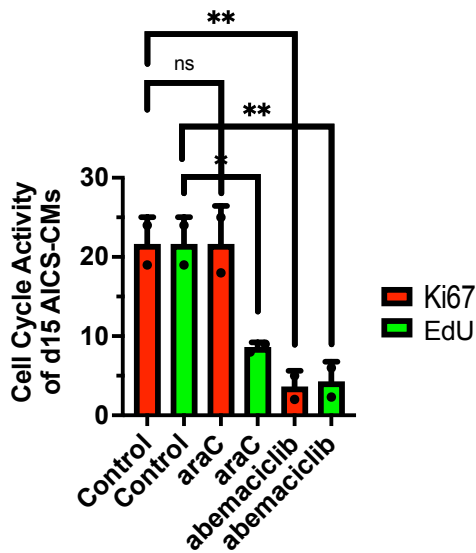

Supplement: Fig. S3 — Analysis of cell cycle inhibitors in d15 hiPSC-CMs. (A) Representative images of Ki67-positive and EdU-positive d15 AICS-CMs treated with either araC or abemaciclib. (B) Quantitation of d15 AICS-CM cell cycle activity. Yellow scale bars = 10 μm. Data are presented as +/− SEM. *P < 0.05, **P < 0.005, ns = not significant. n.s. Statistics were determined using a 2-tailed, unpaired Student's t test. Results are from 3 independent experiments from 2 independent differentiations. [file mmc4.pdf]

Supplemental Figure 4

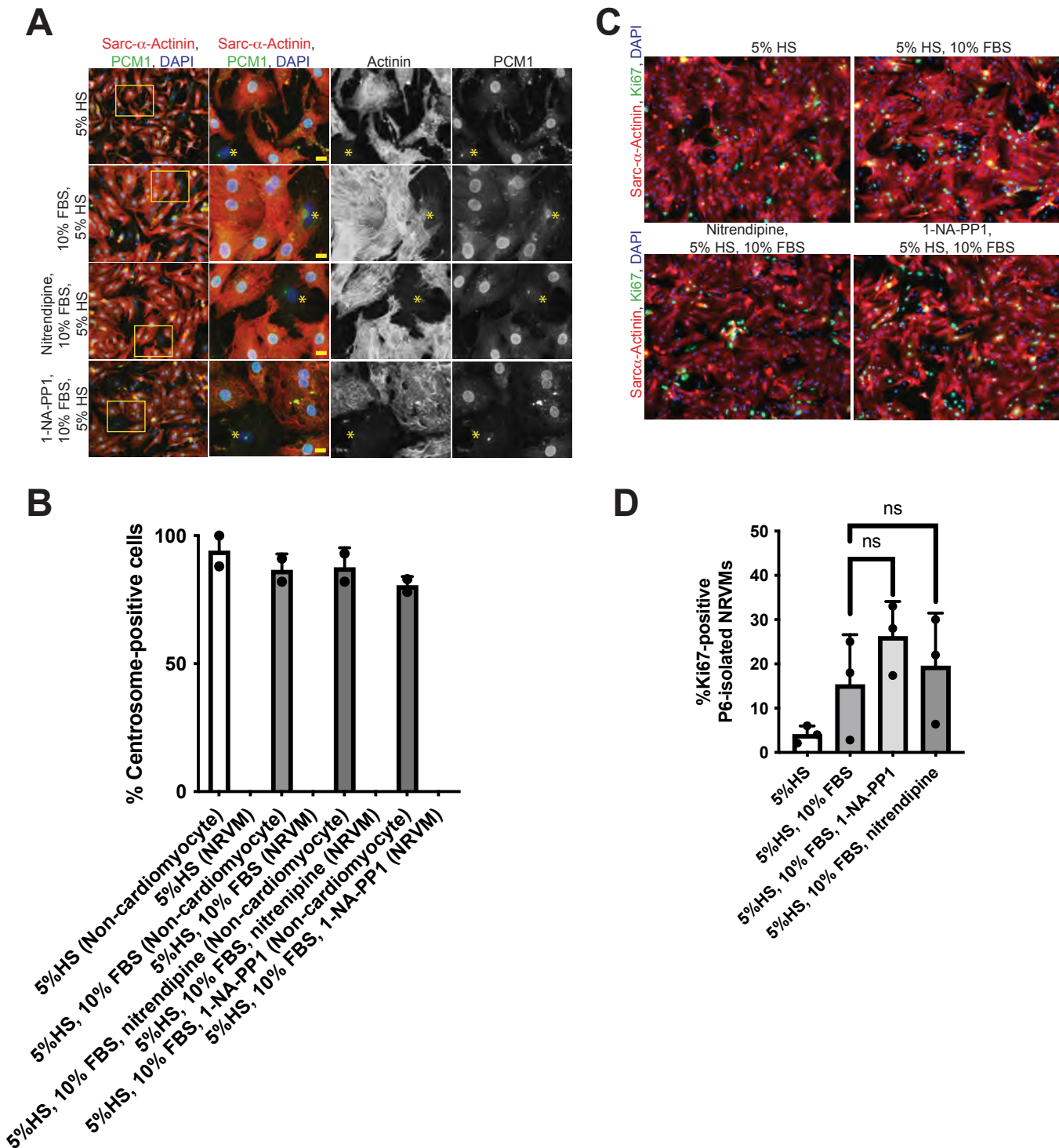

Supplement: Fig. S4 — Analysis of centrosome reassembly and cell cycle activity in NRVMs treated with either nitrendipine or 1-NA-PP1. (A) Representative images of cultured cells isolated from P6 rat hearts. NRVMs, which represent >97 % of the culture, are DAPI positive and Sarcomeric alpha actinin positive. Non-cardiomyocytes (e.g. cardiac fibroblasts), which represent <3 % of the culture, are DAPI positive and Sarcomeric alpha-actinin negative. Yellow asterisks indicate centrosome-positive cardiac fibroblasts. (B) Quantitation of centrosome-positive and centrosome-negative NRVMs and non-cardiomyocytes (e.g. cardiac fibroblasts). (C) Representative images of NRVMs in Ki67-expression assay. (D) Quantitation of Ki67 positive NRVMs. n = total cells scored, 2 independent experiments (B) and 3 independent experiments (D). Data are presented as +/− SEM. ns = not significant. Statistics were determined using a 1-way ANOVA followed by Tukey's test. [file mmc5.pdf]

## Supplemental Figure 5

A

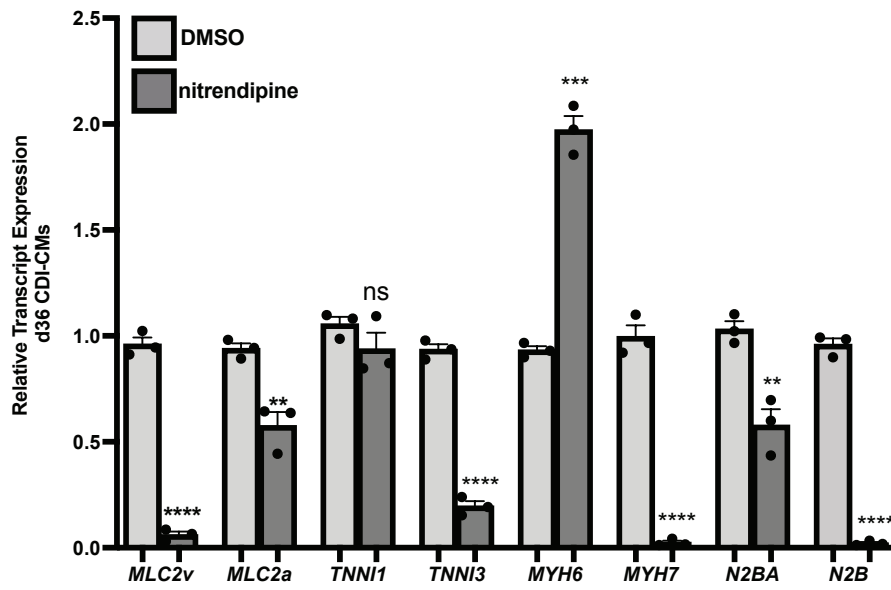

B

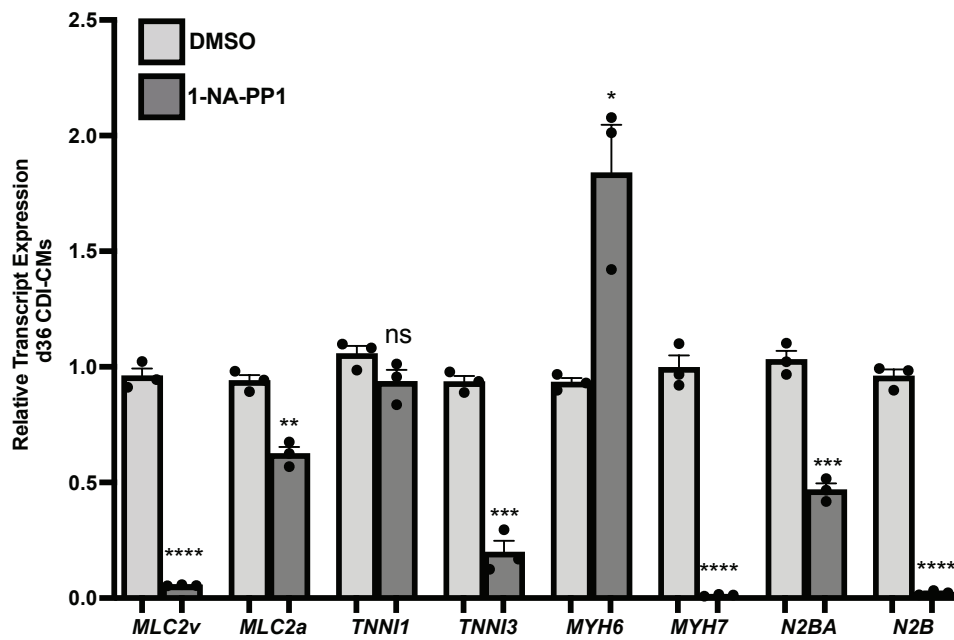

Supplement: Fig. S5 — Sarcomere gene expression in hiPSC-CMs treated with nitrendipine or 1-NA-PP1. (A) qRT-PCR analysis of sarcomere gene expression in d36 CDI-CMs treated with nitrendipine. Results from 3 independent experiments. (B) qRT-PCR analysis of sarcomere gene expression in d36 CDI-CMs treated with NA-PP1. Results from 3 independent experiments. Data are presented as +/− SEM. *P < 0.05, **P < 0.005, ***P < 0.0005, ****P < 0.00005, ns = not significant. Statistics were determined using a 2-tailed, unpaired Student's t test. [file mmc6.pdf]
